# Testing Behavioral Nudges and Prompts in Digital Courses for the Self-guided Treatment of Depression and Anxiety: Protocol for a 3-Arm Randomized Controlled Trial

**DOI:** 10.2196/37231

**Published:** 2022-08-15

**Authors:** Renante Rondina, Trevor van Mierlo, Rachel Fournier

**Affiliations:** 1 Rotman School of Mangement, University of Toronto Toronto, ON Canada; 2 Evolution Health Systems Toronto, ON Canada

**Keywords:** behavioral economics, digital health, attrition, engagement, nudges, mood disorder, anxiety, depression, depressive disorder, mental health, nudge, prompt, behavior change, randomized controlled trial, present bias tip, future gain tip, health platform, mental illness

## Abstract

**Background:**

Despite showing strong evidence of positive outcomes, a common problem in the field of digital health is poor engagement and adherence. Non–health care, for-profit digital ventures, such as Facebook, LinkedIn, and Twitter, conduct behavioral experiments to increase user engagement. To our knowledge, digital health organizations have not published similar types of experiments in ad libitum environments, and there are limited published data indicating whether nudges and prompts can be leveraged to increase engagement with digital health interventions.

**Objective:**

The main objective of our 3-arm randomized controlled trial is to test whether registered members in two well-established digital health courses for anxiety and depression will engage with four different types of nudges and prompts, and whether engaging with nudges and prompts increases engagement within the courses.

**Methods:**

New members who register for the self-guided anxiety and depression courses on the Evolution Health platform will be randomized into 1 of 3 arms. The first control arm will feature a member home page without any behavioral nudges or prompts. The second arm will feature a member home page with a *Tip-of-the-Day* section containing directive content. Arm 3 will feature a member home page with a *Tip-of-the-Day* section containing social proof and present bias content. The third arm will also feature a to-do item checklist.

**Results:**

The experiment was designed in August 2021 and was launched in November 2021. Initially, we will measure engagement with the tips and the to-do checklist by calculating the frequency of use by age and gender. If members do engage, we will then, according to age and gender, examine whether nudges and prompts result in higher course completion rates and whether specific types of prompts and nudges are more popular than others.

**Conclusions:**

Our 3-arm randomized controlled trial will be the first to compare four distinct types of behavioral prompts and nudges in two self-guided digital health courses that were designed to treat mental health issues. We expect the results to generate insights into which types of behavioral prompts and nudges work best in the population. If they are shown to increase engagement, the insights will then be used to apply prompts and nudges to the platform’s addiction-focused courses. Based on the results of the experiment, the insights will be applied to using artificial intelligence to train the platform to recognize different usage patterns and provide specific engagement recommendations to stratified users.

**International Registered Report Identifier (IRRID):**

DERR1-10.2196/37231

## Introduction

### Background

From its inception in the mid-1990s, digital health promised personalized treatments that patients could access from home. It was anticipated that such treatments would have a broad reach, resulting in improved health outcomes and decreased costs [1-3]. Over the past 2 decades, research examining the efficacy of self-guided digital health interventions has intensified. These studies have shown evidence of efficacy, especially for individuals with mental health concerns [4-6].

Although digital health interventions appear to be effective, patterns that have remained consistent in research are poor adherence and a lack of compliance [7-9]. These patterns were first recognized in 2005 and deemed *The Law of Attrition* [10].

As early as 2009, systematic reviews have identified poor adherence and a lack of compliance as an issue that needs to be addressed [11]. This issue persists in a recent meta-analytic review on digital interventions for depression that illustrate efficacy but highlight compliance as a major challenge [12].

This issue of adherence and compliance is complex and is rooted in a number of systemic and individual factors [13-18]. However, it is an important topic, as evidence indicates that higher levels of engagement are associated with improved health outcomes [19,20].

Digital health interventions are becoming increasingly common and accessible. Patients’ use of and trust in these interventions have been intensified by the COVID-19 pandemic. The use of digital health interventions for mental health concerns is growing [21,22], and there is a shortage of professionals that can meet this growing demand. As such, it is important to determine how to increase engagement in digital health programs to maximize their efficacy.

### Behavioral Economics

Behavioral economics leverages psychological experimentation to develop theories about human decision-making, and this field has identified a range of biases around the way people think and feel [23,24].

The utility of behavioral economics is vast, and digital health has leveraged the discipline, allowing researchers to investigate how people use digital health programs and obtain insights on the characteristics of people who use them. Several digital health studies have investigated the use of several strategies, including the use of cooperative games and incentives [25], gamification [26,27], serious games [28,29], and positive behavioral support [30,31].

### Our Use of Behavioral Economics

In our study, we will be examining the effectiveness of nudge theory and behavioral prompts in two ad libitum, self-guided digital behavior change courses.

#### Nudge Theory

Nudge theory, which was popularized in the 2008 book *Nudge: Improving Decisions About Health, Wealth, and Happiness* [24], leverages indirect, positive suggestions to influence decision-making and behavior.

There is a paucity of quality research that analyzes the use of nudges in digital health. A 2019 scoping review examined the use of nudges in both web-based and real-world physical activity interventions [32]. In the 35 publications reviewed, 8 were web-based studies. The authors concluded that although nudging may be an effective approach to promoting physical activity, there are large gaps in research, and further studies are needed that are explicitly based on nudge insights.

A 2020 editorial in *Personalized Medicine* addressed the meaningful adoption of nudges in digital health [33]. The authors acknowledged that the use of nudges in digital health interventions is rare and advocated for the use of nudges to promote positive behavior change.

#### Behavioral Prompts

In applied behavioral analysis, behavioral prompts are cues that are specifically designed to encourage individuals to perform a specific task [34]. In our study, we will be employing the following two types of behavioral prompts, which are anchored in nudge theory: daily tips and a to-do checklist (Table 1).

**Figure 1:**
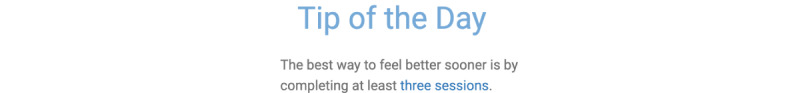
Present bias tip.

Figure 1 is an example of a present bias tip that could randomly appear on a member’s main home page.

**Table 1:** Example nudges and prompts.

Delivery format	Content type	Text example from our study
Tip	Directive content	“Express yourself by uploading your own image!”
Tip	Social proof	“Many members have similar goals as yours. Reviewing other members' goals can help you reach your own.”
Tip	Present bias	“Feel better sooner by learning from others. Read what others have posted on the community.”
Prompt	To-do checklist	“Watch the Getting Started Video”

We have not observed sufficient evidence for determining whether nudges and behavioral prompts can be strategically applied to increase engagement and decrease attrition in courses for depression and anxiety [35]. This is hypothesized in digital health that greater overall engagement may lead to better health outcomes [19,20,36].

Engagement experiments in popular, non–health care digital platforms are common. Although they are scientific in nature, they are not typically published. This makes sense, as they are conducted within private companies and are associated with trade secrets. For example, social network sites, such as Facebook, LinkedIn, and Twitter, generate revenue based on page views and the time users spend on the site. In a 2015 presentation, it was revealed that LinkedIn has over 400 controlled experiments being conducted per day [37]. Similar studies with an ad libitum population are required in digital health, and our study is an attempt to fill this gap.

### Objective

The main objective of our 3-arm randomized controlled trial is to test whether registered members in two well-established, self-guided digital health courses for anxiety and depression will engage with four different types of nudges and prompts, and whether engaging with nudges and prompts increases engagement within the courses.

## Methods

### Digital Health Platform

The digital health platform that will be used in the study is managed by Evolution Health—an evidence-based digital health content provider that features courses based on behavior change techniques, including cognitive behavioral therapy, stages of change, structured relapse prevention, harm reduction, and quizzes based on brief intervention.

The platform offers interactive courses and quizzes for mental health issues, addiction issues, and obesity. The platform contains a moderated community that is based on social cognitive theory.

Memberships are available to individuals who register through the organization’s free-to-consumer program, as well as white-label versions that are licensed by employers, insurance companies, employee assistance programs, educational institutions, nonprofit organizations, for-profit health care organizations, and individual therapists.

### The Interventions

The two interventions in the study contain self-guided, interactive behavior change treatment courses based on state-of-the-art best practices, and both have been examined extensively in the literature [8,38-46].

In the literature, the Overcoming Depression course was previously known as *The Depression Center*, and the Overcoming Anxiety course was known as *The Panic Center*. Both had separate URLs. In 2019, they were each placed onto a single platform, along with other Evolution Health courses and brief interventions.

The two interventions have undergone several iterations since their onset. For example, The Panic Center is the first intervention noted in Eysenbach’s [10] *The Law of Attrition* article. In that iteration, the course contained a tunnel design with 12 successive sessions. The course now has a gamified, free-form matrix design.

Table 2 outlines each course’s current theoretical constructs and evidence base, and Table 3 outlines the main course components.

**Table 2:** Theoretical constructs and evidence base.

Theoretical construct	Overcoming Depression course	Overcoming Anxiety course
Brief intervention	✓	✓
Cognitive behavioral therapy	✓	✓
Gamification	✓	✓
Health belief model	✓	✓
Motivational interviewing	✓	✓
Social cognitive theory	✓	✓
Targeting and tailoring	✓	✓

**Table 3:** Main course components.

Course component	Overcoming Depression course	Overcoming Anxiety course
Avatar upload	✓	✓
Course completion certificate	✓	✓
Course worksheets	✓	✓
Gamified cognitive behavioral therapy course	✓	✓
*Getting Started* video	✓	✓
Goals exercise	✓	✓
Moderated community	✓	✓
Private messaging	✓	✓
Statistics extranet (for corporate clients)	✓	✓
Tailored depression and anxiety test	✓	✓
Therapist extranet	✓	✓

### Ethical Considerations

All data collection policies and procedures adhere to international privacy guidelines [47-49]. At registration, all members tick a checkbox to confirm that they consent to having their data used for research purposes and approve of the platform’s privacy policy.

The platform does not collect personally identifiable information except a user’s email address, which is required for registration confirmation and the retrieval of lost passwords. Email addresses are encrypted in a separate database and are not included in data reports.

### Power and Sample Size

Our goal is to obtain 0.95 power to detect a significant difference in the proportion of users who complete at least 1 cognitive behavioral therapy session between the two treatment groups (arm 2 vs arm 3). Based on a preliminary analysis of an early subsample of users from November 1 to December 9, 2021 (arm 1: n=510; power=3.14%; arm 2: n=484; power=5.79%) and a conventional α level (.05) for statistical significance, we would require a sample of 4224 users, with 1414 users in each treatment group. Multimedia Appendix 1 shows our study’s CONSORT-EHEALTH (Consolidated Standards of Reporting Trials of Electronic and Mobile Health Applications and Online Telehealth) checklist [50].

### Randomization

**Figure 2:**
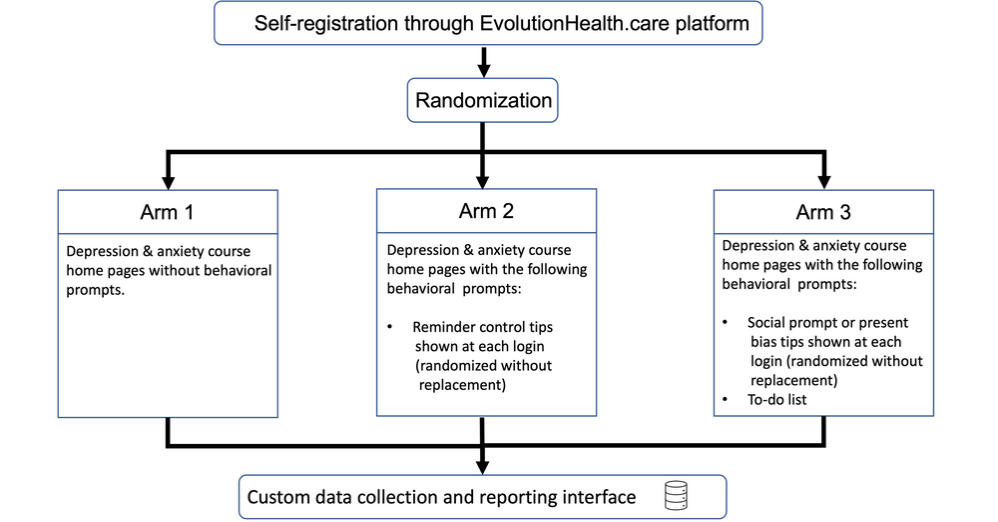
Study flow.

During the registration process, by using a random number generator, new members will be assigned into 1 of the 3 arms (Figure 2). Randomization will be conducted by using simple randomization.

### Intervention Groups

**Figure 3:**
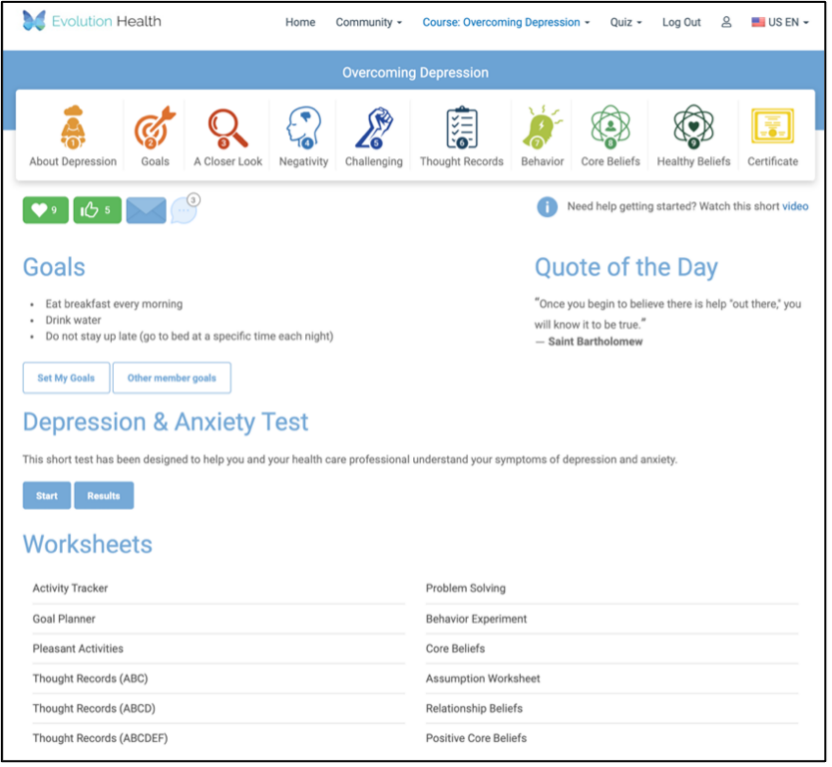
Member home page for arm 1.

Members allocated to the first arm will be presented with a member home page that contains no behavioral prompts. Figure 3 is a screenshot of an arm 1 homepage for a member who chooses to engage with the depression course.

**Figure 4:**
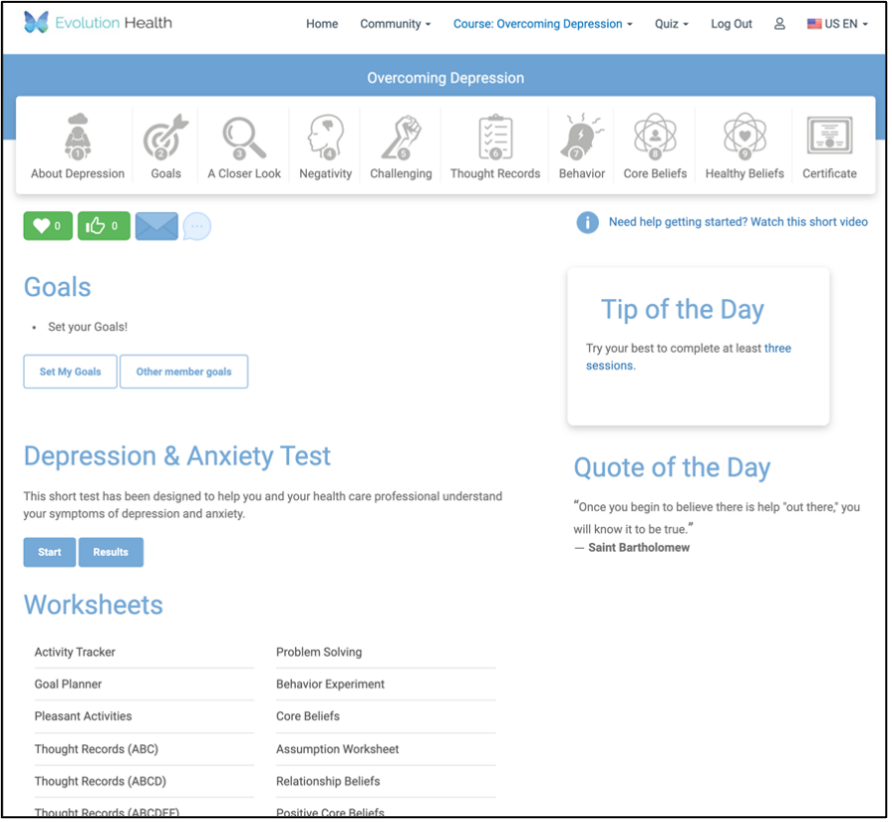
Member home page for arm 2.

Members allocated to the second arm will be presented with a member page that contains a *Tip-of-the-Day* section containing directive content. The randomization strategy for the 35 directive tips is randomization without replacement. Figure 4 is a screenshot of an arm 2 homepage for a member who chooses to engage with the depression course.

Members allocated to the third arm will be presented with two sections that contain interactive prompts. The first is a *Tip-of-the-Day* section containing social proof and present bias content. At each log in, members will see a new tip. The randomization strategy for the tips is randomization without replacement. There are 15 social proof tips and 15 present bias tips.

**Figure 5:**
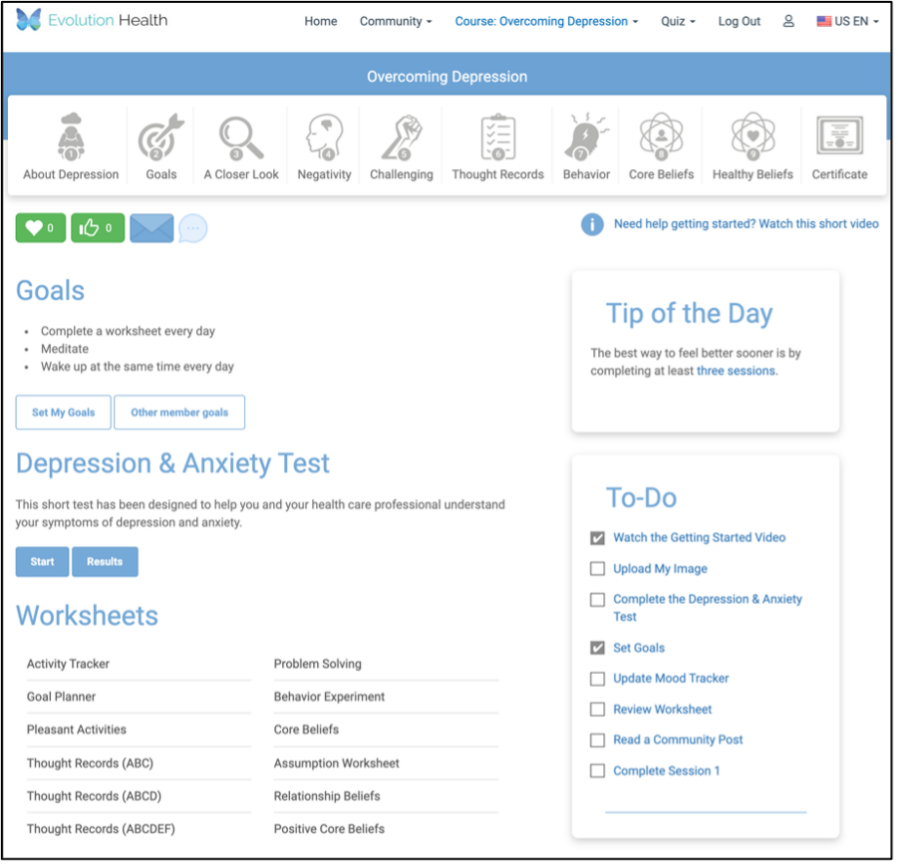
Member home page for arm 3.

In addition to the tips of the day, the third arm will also feature a to-do checklist that lists interactive course components. When a member clicks on an interactive component, they will be brought to the exercise. When they complete the exercise, the item is marked as complete with a check mark. Figure 5 is a screenshot of an arm 3 homepage for a member who chooses to engage with the depression course.

### Data Collection

Evolution Health developed a custom data collection interface and reporting mechanism. Data will be collected for each member who is randomized into the experiment. Data on age and gender will be collected at registration or at secure sign-on in various white-label versions. The course components that will be promoted by the tips and to-do items are listed in Table 4.

The following behaviors are tracked in the custom database for each tip and to-do item that is randomly presented to a user: whether a tip was shown, whether a tip was clicked on, and whether a user completed the course component described in a tip or to-do item.

It is possible for a single member to participate in both courses. However, the study was designed to test behavioral economics prompts, not courses. Participants will be randomized to an intervention arm in which behavioral nudges and prompts are consistent across courses.

**Table 4:** Course components tracked for engagement.

Action code	Course component
1^a^	Uploading a personal image to their profile
2^a^	Completing cognitive behavioral therapy session 1
3^a^	Use of the program’s diary
4^a^	Read a community post
5	Review another member’s profile
6	Posting in the community
7^a^	Review a course worksheet
8^a^	Set personal goals
9	Read other members’ goals
10^a^	Complete the depression and anxiety test
11^a^	Watch the *Getting Started* video
12	Give a community member a thumbs-up
13	Encourage a community member by clicking their “show support” icon
14	Private message a community moderator

^a^Item in the to-do checklist.

### Primary Outcome: Engagement With Tips and To-do Items

We will first measure engagement with the tips and to-do items in arms 2 and 3. We will compare the frequency of engagement with each nudge and behavioral prompt by age and gender. A member’s engagement with tips will be measured as the proportion of tips that were clicked. Specifically, it will be a value between 0 and 1, calculated as the number of tips that a member clicked divided by the number of tips that were presented to the member. Engagement with to-do items will be measured as the number of to-do items that a member clicked, and course component completion rates will be measured as the number of course components that a member completed. All comparisons between arms 2 and 3 will be conducted by using an independent sample 1-tailed *t* test; a conventional α level (.05) for statistical significance will be used.

### Secondary Outcomes: Completion of Course Components

A secondary outcome of this experiment is to test whether the presentation of tips and to-do items increases the overall completion of course content. We will achieve this by comparing course component completion rates in arm 2 and arm 3 via a 1-way ANOVA in which the types of tips are used as the independent variable. We will then assess if age and gender predict which type of tip is the most engaging by using multiple regression.

### Tertiary Outcomes: Engagement With Types of Tips

Another outcome of this experiment is to determine what types of tips are most engaging (directive, social proof, or present bias tips). We will assess this by comparing engagement with the tips in arm 2 and arm 3 via a 1-way ANOVA in which the types of tips are used as the independent variable. We will then assess if age and gender predict which type of tip is the most engaging by using multiple regression.

## Results

This protocol was originally designed in August 2021. Alpha and beta testing on Evolution Health’s staging environment began in October 2021. The protocol was revised in October 2021, and the experiment was pushed live in November 2021. We exceeded the sample size requirements in each arm and concluded data collection in May 2022.

## Discussion

### Hypotheses

We hypothesize that members will engage with the prompts and nudges. We also hypothesize that the secondary results will generate insights on the types of prompts and nudges that are the most likely to result in engagement among members. This finding may be important, as dose-response relationships have been observed in digital health interventions [8,51].

### Practical Implications

If we learn that the prompts are used by members, the arm 2 or arm 3 home page will become the default user home page for all new and existing members.

### Strengths and Limitations

A strength of this experiment is that it will be conducted in an ad libitum environment. Unlike most digital health studies, ours will not be conducted with a small population in a controlled environment. Further, members will not be aware of the experiment, which will limit participant bias and the Hawthorne effect.

A limitation of this experiment is that, especially due to the anonymity of members, we have no way of knowing who members are. We have no way of knowing whether registrants are people with depression or anxiety who are seeking help, or are browsing for other reasons. However, we have exceeded sample size requirements and believe that the large number of subjects should dampen these effects on the results.

A final limitation is that not all white-label clients license all course components. For example, some community tools and the depression and anxiety test are feature flags that are not used by all white-label clients. As the primary purpose of this experiment is to test whether members engage with prompts and nudges, we do not expect this particular limitation to hamper the overall results.

### Future Directions

Although the content is different, the platform’s addiction courses have user home page interfaces that have the exact same layout as that of the arm 1 home page interface. If the results from our study indicate that members engage with the prompts and nudges in arm 2 and arm 3, we will replicate this experiment with new members who register for these courses. Although the addiction courses focus on problem drinking and smoking cessation, the use of nudges and prompts to increase engagement may be generalizable to many digital health courses that focus on addictions and mental health.

The secondary and tertiary outcomes will help shape the development of future nudges and prompts. If specific types of nudges and prompts are appealing to specific gender and demographic groups, we will customize the content for these groups and analyze their effectiveness in future studies.

We are currently employing natural language processing to analyze identifiable contexts (ie, change talk) in our communities [52]. We will apply the findings from that experiment to the member-generated content from the goals and diary tools to better determine the types of prompts and nudges that result in engagement among members. The overall goal will be to use artificial intelligence to train the platform to recognize different usability patterns and show specific engagement prompts and nudges to stratified populations.

The goal of the courses is to promote wellness and increase access to efficacious, self-guided support for people with depression and anxiety. The focus of the study is restricted to testing whether nudges and prompts increase engagement. If the study is successful, future research will need to determine if nudges and prompts contribute to decreases in depression and the frequency and intensity of panic attacks.

## Appendix

Multimedia Appendix 1 CONSORT-eHEALTH checklist (V 1.6.1).

## Abbreviations

CONSORT-EHEALTH: Consolidated Standards of Reporting Trials of Electronic and Mobile Health Applications and Online Telehealth

## Data Availability

Allowing the data set from our study to be publicly available would violate the platform’s data privacy policy, terms of use, and user agreement. However, researchers interested in accessing the data set or other platform data sets for noncommercial purposes are encouraged to contact Evolution Health.
